# The Distribution of True Visual Field Progression Rates in Glaucoma

**DOI:** 10.1167/tvst.13.9.31

**Published:** 2024-09-30

**Authors:** Andrew Anderson

**Affiliations:** 1 Department of Optometry & Vision Sciences, The University of Melbourne, Australia. e-mail: aaj@unimelb.edu.au

I congratulate Montesano et al.^1^ on their recent article. I believe their methodological approach represents a significant and—importantly—useful advance on previous work attempting to establish the “true” underlying distribution of glaucomatous progression rates, separate from such factors as measurement noise.^2^

Montesano et al.^1^ assume that visual field sensitivity can only worsen or remain unchanged, and so true progression rates cannot be positive. Although this might be true for absolute measures of visual sensitivity, the authors’ model data for the age-corrected summary index, mean deviation (MD). It seems reasonable to assume that the true rate of absolute visual sensitivity change with ageing will vary among people. One might expect around half of people without progressive disease to show a slight positive change in their true MD over time, simply because their visual system loses sensitivity at a rate less than that predicted by the age correction. Therefore the assumption that the true rate of disease progression can be modeled by an exponential distribution that can take only zero or negative values may not strictly hold. Given this, their fitted exponential functions may not perfectly describe the true progression rate. Rather, some of this true rate would also influence parameters describing the Gaussian “noise” component.

## References

[1] Montesano G, Crabb DP, Wright DM, Rabiolo A, Ometto G, Garway-Heath DF. Estimating the distribution of true rates of visual field progression in glaucoma*.* *Transl Vis Sci Technol*. 2024; 13(4): 15. 10.1167/tvst.13.4.15 PMC11008752 38591945

[2] Anderson AJ. Estimating the true distribution of visual field progression rates in glaucoma*.* *Invest Ophthalmol Vis Sci*. 2015; 56: 1603–1608. 25678691 10.1167/iovs.14-16329

